# Healthy memory aging - the benefits of regular daily activities increase with age

**DOI:** 10.18632/aging.203753

**Published:** 2021-12-16

**Authors:** Olga Krakovska, Gregory J. Christie, Faranak Farzan, Andrew Sixsmith, Martin Ester, Sylvain Moreno

**Affiliations:** 1 Department of Gerontology, Simon Fraser University, Vancouver, BC, Canada; 2 School of Computer Science, Simon Fraser University, Burnaby, BC, Canada; 3 The School of Interactive Arts and Technology, Simon Fraser University, Surrey, BC, Canada; 4 AGE-WELL National Innovation Hub: Digital Health Circle, Surrey, BC, Canada; 5 Centre for Engineering-led Brain Research (eBrain Lab), Surrey, BC, Canada; 6 School of Mechatronic Systems Engineering, Simon Fraser University, Surrey, BC, Canada

**Keywords:** healthy aging, cognitive decline, daily activities, machine learning, big data

## Abstract

As the number of older adults increases, so does the pressure on health care systems due to age-related disorders. Attempts to reduce cognitive decline have focused on individual interventions such as exercise or diet, with limited success. This study adopted a different approach by investigating the impact of combined daily activities on memory decline. We used data from the National Institute of Aging’s Health and Retirement Study to explore two new questions: does combining activities affect memory decline, and if yes, does this impact change across the lifespan? We created a new machine learning model using 33 daily activities and involving 3210 participants. Our results showed that the effect of combined activities on memory decline was stronger than any individual activity’s impact. Moreover, this effect increased with age, whereas the importance of historical factors such as education, and baseline memory decreased. The present findings point out the importance of selecting multiple, diverse activities for older adults as they age. These results could have a significant impact on aging health policies promoting new programs such as social prescribing.

## INTRODUCTION

Population aging is one of the significant challenges of the 21st century as the number of adults over the age of 65 continues to increase. This shift in demographics represents a substantial challenge to healthcare systems, as older adults are significantly more at risk of developing dementia and other neurodegenerative disorders [1]. Aging and cognitive decline are closely correlated. For example, median scores in the Mini-Mental State Examination (MMSE), a widely used clinical assessment for dementia, decline by approximately 10% from age 50 to 85 and up to 35% for the lowest-scoring quartile [2]. At present, we have few solutions to deal with this growing health issue with most studies focussing on specific interventions such as drugs or training programs with limited success.

Despite the clear trend for cognitive decline with aging, there is significant inter-personal variance. The reasons for this variance are not entirely understood but are suggested to be strongly dependent upon earlier lifetime experiences [3]. This is often described as ‘cognitive’ or ‘brain’ reserves, which are drawn upon in later life to slow the rate of cognitive and memory decline and to avoid or delay overt symptoms [4]. It is proposed that these experiences create multiple, redundant neural processing networks that are more resistant to disruption due to brain damage and compensatory mechanisms that help create new neural processing networks in response to such damage [5]. Previous research also suggested that these experiences can lead to increased brain size and synapse counts, which limits the magnitude of cognitive impairment until brain damage exceeds a certain critical threshold [6]. There is good evidence that the resulting reserves are influenced by genetics [7] and environmental factors, affecting the reserves in different directions. For example, educational attainment [8] has been shown to influence reserves positively, whereas alcohol consumption [9] and smoking [10] have been shown to influence reserves negatively. A considerable number of studies have investigated the impact of such prior life factors in later life, with a common finding that they typically contribute significantly to cognitive health and, thus, improve cognitive performance in aging. Although the effects of these genetic and historical life factors are considered robust, they offer little hope to current older adults because they cannot be altered in later life. What is promising and can be modified is the effect of daily activities on maintaining cognitive health and memory as adults age. Indeed, several studies have attempted to determine whether diet [11], cognitive training [11], physical exercise [12], music practice [13], meditation [14], social engagement [15, 16] or combined interventions [17] are effective at slowing cognitive decline due to aging. However, these studies have typically examined the benefits of these activities individually and the overall evidence for their benefit is inconsistent [18]. Furthermore, conclusions have been limited due to small effect sizes, low numbers of participants, healthy survivor bias, self-selection into studies, limited follow-up measurements, lack of control groups, and lack of combined interventions. As a result, we are still left with the question of ‘what can older adults do now to maintain their cognitive performance?’ Our approach is to investigate the relationship between several potential daily activities and aging memory. This alternative approach has the benefit that it allows us to investigate a broad set of factors in a data-driven manner, rather than focusing on individual factors studied in isolation and with typically low numbers of participants.

In this study, we created a new combined analysis method based on machine learning, which allowed us to investigate a large population of older adults at varying ages. Rather than conducting intervention trial studies, our investigation exploited the natural person-to-person variability in their daily activities and assessed how they affected memory at different ages in real world settings. This unique and powerful methodological approach allowed us to identify, quantify and understand how engagement in numerous key activities is related to cognitive health and to provide older adults with information that they can immediately act upon. Finally, this analysis provides a unique ability to identify the dynamics of the relationship between memory, background factors (education etc.) and daily activities, and how this relationship evolves as an individual ages.

## RESULTS

### Selection of daily activities

Feature selection analyses identified 17 daily activities out of 33 that were significantly associated with changes in cognition, for at least one age group, by at least one method. These 17 variables represented cognitively challenging, physical, or social activities. Table 1 lists the selected variables with corresponding *p*-values for each age category. Notably, no single daily activity was significantly associated with all age groups. Only one daily activity (*use computer*) was identified as associated with changes in memory in four age groups. Only four daily activities (*do activities with grandchildren* and *do word games, meet up with children,* and *speak on phone with other family members*) were associated with changes in memory in two age groups. The rest of daily activities were significantly associated with changes in memory in only one age group.

**Table 1:** List of daily activities, which were found to be significantly associated with changes in memory, at least in one age category, at least by one method.

**Daily Activity**	**Age Group**				
	**65–69**	**70–74**	**75–79**	**80–84**	**85–89**
DO ACTIVITIES WITH GRANDCHILDREN	.30	**.05**	.33	**<.01**	.11
VOLUNTEER YOUTH	.54	.13	.39	**.03**	.63
ATTEND SPORTS/SOCIAL/CLUB	**.02**	.32	.48	.59	.21
READ	**.03**	.14	.26	.17	.13
DO WORD GAMES	.25	**.05**	**.05**	.14	.90
PLAY CARDS AND GAMES	.28	.21	.68	.16	**.01**
USE COMPUTER	**<.01**	**<.01**	**.04**	**.01**	.27
BAKE OR COOK	.13	.15	.73	**.04**	.45
SEW OR KNIT	**.02**	.52	.75	.15	.26
WALK FOR 20 MINS	.61	.33	.52	**.04**	.39
MEET UP WITH CHILDREN	.17	**<.01**	.51	**.05**	.39
SPEAK ON PHONE WITH CHILDREN	.55	.19	**.03**	.10	.85
WRITE OR EMAIL CHILDREN	.16	.30	.11	.44	**<.01**
SPEAK ON PHONE WITH OTHER FAMILY MEMBERS	.38	**.02**	**.01**	.43	.15
WRITE OR EMAIL OTHER FAMILY MEMBERS	**.05**	.06	.11	.19	.51
SPEAK ON PHONE WITH FRIENDS	.26	.06	**.02**	.74	.19
WRITE OR EMAIL FRIENDS	**.05**	.38	.14	.06	.19

### Importance of individual daily activities

**Figure 1:**
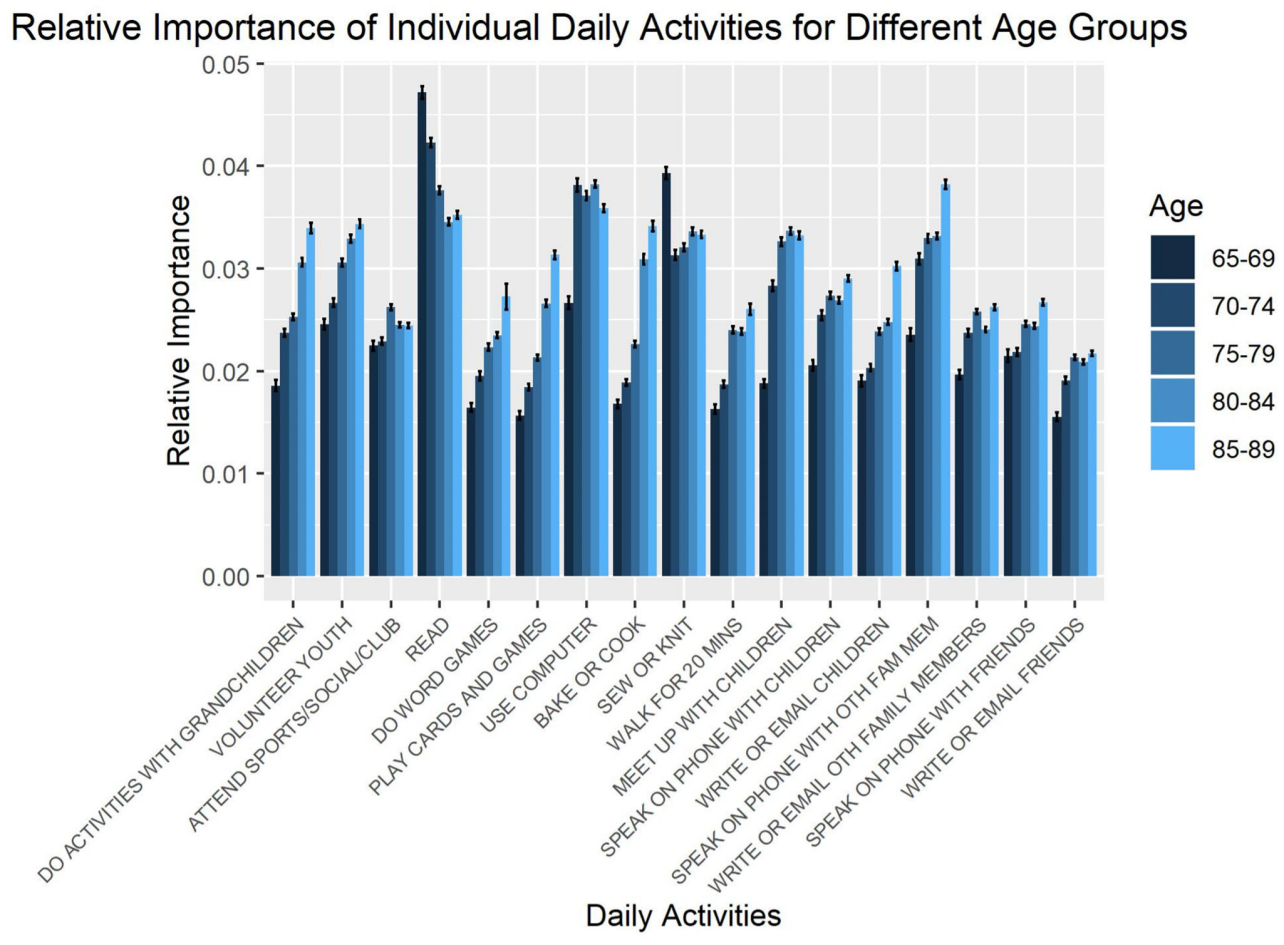
**Relative importance of daily activities for predicting changes in memory, as estimated from sensitivity analysis.** Error bars represent standard errors across repetitions in the sensitivity analysis.

The relative importance of daily activities at predicting memory changes was quantified through sensitivity analyses for each age group. Figure 1 shows the relative importance of individual daily activities for each age group. No clear pattern of age-related changes in the relative importance of individual daily activities for predicting cognition changes was identified.

### The combined effect of daily activities on memory

**Figure 2:**
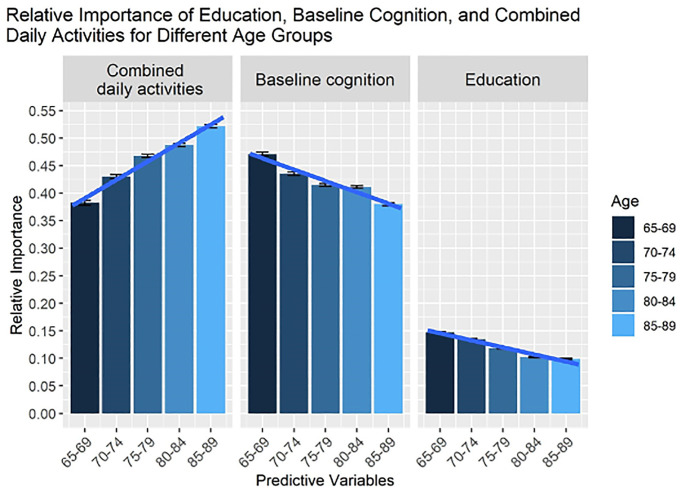
**The relative importance of all life activities, education, and baseline memory for predicting changes in memory, as estimated from sensitivity analysis.** In contrast to Figure 1, we show the combined importance of life activities (total sum). Note that on average age-related changes are characterized as an increasing trend for the combined effect of life activities on changes in cognitive health, whereas effects of education and baseline cognition are decreasing.

The relative importance of daily activities from Figure 1 were combined for each age group. Figure 2 shows the relative importance of these combined daily activities compared with education and baseline cognition for each age group, demonstrating their power in predicting memory changes. The relative importance of the combined daily activities was computed as an arithmetic sum of the individual relative importance. Although finding regular patterns in the predictive power of individual daily activities was elusive, clear trends could be observed by considering their combined effects.

### The importance of daily activities increases with age

As can be seen from Figure 2, the relative importance of combined daily activities on memory increased with age, which was confirmed by ANOVA (*p* < 0.001). Furthermore, pairwise comparisons confirmed significant differences in the relative importance of combined daily activities between age groups with *p* < 0.0002 for all pairs. In contrast to the combined effects of daily activities, the importance of education and baseline cognition on memory decreased with age (see Figure 2), which was confirmed by ANOVA (*p* < 0.001). Pairwise comparisons also showed significant differences between age groups (*p* < 0.001) except the 75–79 and 80–84 age groups for baseline cognition and 80–84 and 85–89 age groups for education were not significantly different.

## DISCUSSION

Current health and medical literatures provide inconclusive information on what factors can impact aging memory, leading to confusion amongst both medical and general communities. Some of this confusion could be explained by methodological limitations such as a low number of study participants, or the complexity of measuring the impact of daily activities across a lifespan. To avoid these limitations, this study used the extensive database of the Health Retirement Study [19] and created machine-learning analyses to investigate the relationship between daily activities (identified from literature) and aging memory, controlling for robust factors such as level of education and baseline memory.

Our analyses replicated the results of previous studies that showed baseline cognition and memory health [20] and level of education [8] are both critical factors that influence the rate of memory decline. Such studies found a moderate association between the rate of memory change and baseline memory, which suggests that changes in memory reflect person-specific factors and are not an inevitable result of the aging process. A novel aspect of our results is the confirmation of these findings using real-life context data and much larger sample size.

To further explore whether other factors influence aging memory, this study was extended to investigate the impact of several daily activities. Several previous studies have reported a positive relationship between some daily activities such as physical activity [12] or music [13] on the cognitive aging process. Our findings confirm that the contribution of individual daily activities for predicting changes in memory is significant but relatively small. Specifically, we estimate that the relative, quantitative importance of individual daily activities does not exceed a 10% change in the baseline memory. This result is consistent with those from previous studies, in which the effect of physical interventions on cognitive functioning was similarly estimated to be around 10% and independent of the length of those interventions [21, 22]. These small effects may also explain the conflicting or inconsistent findings in the literature and the limitation of focusing on the benefits of a single activity.

A potential limit could be the interplay between ‘self-reported frequency of daily activities’ and more objective values such as ‘hour per month’. This relationship could be complex. However, this issue was addressed in previous studies and their results showed that self-reported values, rather than more objective indicators, represent the issue in questions more accurately [23, 24]. Given that older adults do not typically engage in a singular daily activity but in an assortment of them, the combined effects of various daily activities were investigated. The findings of this study clearly showed that the effect of combined, rather than single, daily activities on memory significantly increases with age. Indeed, by the age of 90, the importance of combined daily activities on memory had increased by almost 40%. Interestingly, this age-dependent trend was not apparent when considered separately for individual daily activities. A clear, monotonic pattern of age-related changes in the relative importance of individual daily activities was not observed. More importantly, not a single daily activity was found to be significant for all five age groups. It was only after combining individual daily activities that age-related changes in the importance of daily activities for predicting changes in memory were observed. Although additional studies are required to model the key factors linking daily activities to cognitive benefits in older adults, our findings indicate that, as individuals age, their cognitive health is less influenced by historical factors and is increasingly influenced by more immediate factors such as daily tasks and leisure activities. This suggests that the decline in cognition associated with aging can be reduced by having an individual remain active both physically and mentally.

### Future research and study limitations

While the results of this study are encouraging, the findings are based on a statistical model that uses historical observations relating to daily activities, education and cognition baseline as predicting variables. In addition, the study had two other significant limitations: i) The data set did not include other potentially influential factors such as diet or socioeconomic factors. ii) Causal relationships were not considered. For example, part of the memory baseline may be explained by education, which may ultimately affect the quantitative assessment of relative importance. Similarly, engagement in one daily activity may be directly affected by the engagement in another activity. This relationship between daily activities needs to be understood further to develop causal relationship models in the future. Despite these limitations, and because the considered set of daily activities is unlikely to be highly interconnected, we feel that the results of this present study are valid, but additional studies need to be conducted to confirm our findings substantially.

#### Policy recommendations

Care for patients with dementia is challenging, labor-intensive, and chronic, which generates high costs for health systems. Currently, in the United States, the combined direct and indirect care cost for dementia is estimated to be about $277 billion per year [2], almost certainly increasing in the coming years. Since the 1980s, significant efforts have been made to develop drug treatments for dementia, but these efforts have largely been unsuccessful and current drug-based treatments have little or no efficacy in rehabilitating or reducing the symptoms of dementia. Moreover, the development of drug-based therapies is costly and slow. It means that by the time an effective drug is discovered, validated, and brought to market, a crucial window may have closed in which we could have slowed the onset of dementia and sustained normal cognitive health. Our research suggests that it is possible to improve the well-being of the older adults. This may be achieved by non-pharmacological interventions that promote active aging and engagement in a rich array of daily activities. This would likely have significant benefits in improving the cognitive health of older adults and would reduce demands on health services and family caregivers stemming from dementia and other health conditions.

## METHODS

### Data source

Data obtained through interviews and surveys were taken from the 2008 and 2014 releases of the Health and Retirement Study (HRS), a longitudinal cohort study on health, retirement, and aging [19]. In particular, the study contained information from individuals with a broad range of ages on cognition and daily activities.

### Sample selection

Three thousand five hundred seventy participants were selected based on: i) aged between 65 and 89 years (2014 release); ii) education record (2014 release); iii) memory assessment (2008 and 2014 release); iv) at least one daily activity (2014 release; see next section); and v) having gone through typical testing procedures (i.e., no interruptions, or problems with hearing). The participants were then reduced to 3,210 based by removing those with more than 15% missing data across all variables. Any remaining missing data were replaced with the median across participants, separately for each variable. The selected 3,210 participants were then grouped into five age ranges: 65–69 years (757); 70–74 years (840); 75–79 years (800); 80–84 years (518); and 85–89 years (295). It is worth noting that the upper age limit for the third age category corresponded to the USA’s life expectancy, which is about 79 years of age.

### Measures

#### Baseline memory and memory changes

The goal of our research was to explore the influence of background factors and current daily activities on memory and to examine how the importance of background and current daily activities change with age. Memory was assessed in the HRS by ‘immediate’ and ‘delayed’ word recall tests that are commonly used in dementia diagnosis [25]. Memory changes that often precede cognitive decline are widely used to measure cognitive health in older adults [26, 27]. During the recall test, participants were asked to memorize a list of ten words, which was read aloud by the interviewer [25]. Participants were then asked to recall as many words as possible (immediate recall). After five minutes of answering other questions, participants were then asked again to recall as many words as possible (delayed recall). The *baseline memory* measure was the sum of words correctly remembered during both the immediate and delayed recalls (i.e., ranging between 0 and 20). The *changes in memory* were subsequently defined as the difference between the HRS 2008 and 2014 *baseline memory* measures.

#### Daily activities

33 *daily activities* (see Table 2) were selected from the HRS 2008 database that covered diverse aspects of daily life, including cognitively challenging, mild physical, housework and leisure activities. Daily activities associated with personal hygiene, dressing, eating, maintaining continence were excluded. Participants had been asked to indicate their level of engagement in each activity ranging from ‘never’ or ‘at least once a month’ to ‘several times a month’ up to ‘daily’. Although continuous by design, the daily activities were mapped into six or seven ordered categories.

**Table 2:** List of all daily activities included in our initial data set.

**Number**	**Daily Activity**
1	CARE ADULT
2	DO ACTIVITIES WITH GRANDCHILDREN
3	VOLUNTEER YOUTH
4	CHARITY WORK
5	EDUCATION
6	ATTEND SPORTS/SOCIAL/CLUB
7	ATTEND NON RELIGIOUS ORGS
8	PRAY PRIVATELY
9	READ
10	WATCH TELEVISION
11	DO WORD GAMES
12	PLAY CARDS AND GAMES
13	DO WRITING
14	USE COMPUTER
15	MAINTENANCE/GARDENING
16	BAKE OR COOK
17	SEW OR KNIT
18	DO HOBBY
19	PLAY SPORT/EXERCISE
20	WALK FOR 20 MINS
21	PARTICIPATE COMMUNITY ARTS GRP
22	MEET UP WITH CHILDREN
23	SPEAK ON PHONE WITH CHILDREN
24	WRITE OR EMAIL CHILDREN
25	COMMUNICATE BY SOCIAL MEDIA
26	MEET UP WITH OTHER FAMILY MEMBERS
27	SPEAK ON PHONE WITH OTHER FAMILY MEMBERS
28	WRITE OR EMAIL OTH FAMILY MEMBERS
29	COMMUNICATE BY SOCIAL MEDIA WITH FAMILY MEMBERS
30	MEET UP WITH FRIENDS
31	SPEAK ON PHONE WITH FRIENDS
32	WRITE OR EMAIL FRIENDS
33	COMMUNICATE BY SOCIAL MEDIA WITH FRIENDS

#### Historical factors

The level of education in years from the HRS 2008 database was also included in the analysis as it is considered a well-established measure of cognitive and memory reserve [8].

### Data analyses

Taking into account education, machine learning was used to predict age-related changes in memory from participation in daily activities. Given the growing body of evidence indicating non-linear relationships between daily activities and physical health outcomes, no assumptions were made regarding the relationship between daily activities and potential cognitive outcomes. To that end, a two-step approach was followed to identify and quantify the relationship between daily activities and memory function.

### Step 1. Feature selection to identify relevant predictive variables

In step one, a combination of non-linear techniques (Distance Correlation [28] and Random Forest [29]) was used to identify the most relevant variables with the goal of dimensionality reduction and support vector regression (SVR) for constructing predictive models. It should be noted that the use of SVR was not to focus on predictive accuracy but rather to investigate the importance of each predicting variable in explaining the target variable and exploring how a variable’s importance changes as a function of age.

Still, we estimated the predictive accuracy by randomly splitting dataset into training and testing subsets, with 80% used for training, and 20% used for testing for each age category, and further computing the coefficient of determination for the testing set. We repeated this estimation for 100 times, with mean coefficient of determination being 0.51. Non-linear techniques were considered most appropriate due to their versatile ability to capture functional relationships between variables of interest and that linearity between variables could not be assumed. Besides, non-linear techniques are better at accurately capturing strong associations between non-linearly dependent variables, while retaining the ability to capture strong associations between linearly dependent variables [30].

Distance Correlation is a measure of dependence between two random variables, not necessarily linearly related, which is zero only when variables are independent [28]. The distance correlation between memory changes and the daily activities was computed separately for each age group. The significance of correlations was tested with a bootstrap procedure, based on ~300 samples, as implemented in the R statistical toolbox ‘Energy’ [31].

Random forest is an ensemble learning algorithm that is an extension of decision trees. An algorithm progressively learns to predict the value of a target variable based on several input variables. Random forests are a way of averaging multiple decision trees, trained on subsamples of the same training data, to reduce the variance of the trained model [29]. One advantage of using random forests is the ability to identify, as a batch, groups of predicting variables that are related to the target variable. Random forest regression was used to predict changes in memory based on daily activities, education, and memory baseline for each age group. Each feature’s statistical significance was estimated for each age group using a permutation test based on ~300 samples, as implemented in the *R* package ‘pRF’ [32]. For further analysis, a subset of daily activities, which were found to be associated with *memory* changes at the 95% confidence interval (at least in one age category and by one method), was identified.

### Step 2. Sensitivity analysis to quantify the relative importance of daily activities

The next step was to predict changes in memory using a sub-set of daily activities, which were identified as significant in the previous analysis, as well as education level and baseline cognition from the 2008 HRS release. Each age category was analyzed separately to quantify the relative importance of each predicting variable in its ability to explain the variability of the target variable. This procedure was performed using an SVR (based on a Gaussian kernel with a 10-range grid search for hyper-parameter optimization [33]) in combination with sensitivity analysis. Sensitivity analysis is based on the idea that changes in a relevant feature should yield substantial changes in the target variable. Features (variables) with low sensitivity are considered less important, whereas those with high sensitivity are considered more valuable. A random selection of several training sub-samples of the data with the corresponding responses of the target variable, taking into account the physiological well-being of an individual, was implemented in the R statistical toolbox ‘rminer’ [34]. The measure of importance was defined as a relative measure of dispersion in the target variable caused by changes in a given feature. Therefore, the importance of different features represents a unified measure that can be subsequently summed up.

The sensitivity analysis was repeated 200 times for each age group. As a result, each predicting variable was associated with a distribution of its important values, describing its ability to cause changes in the predicting variable. Analysis of variance (ANOVA - a generalization of the two-sample *t*-test to more than two groups with the null hypothesis that the group means are equal) was applied to test for significance of the differences in a variable’s importance between age groups. To further compare the variable’s importance between age groups, a series of two-sample Tukey Honest Significant Differences tests [35] was performed on a pairwise basis.

### Consent for publication

Corresponding Author has the right to grant on behalf of all authors and does grant on behalf of all authors, an exclusive licence (or nonexclusive for government employees) on a worldwide basis to the BMJ Publishing Group Ltd to permit this article (if accepted) to be published in BMJ editions and any other BMJPGL products and sublicences such use and exploit all subsidiary rights, as set out in our licence.
